# Amyloid and tau signatures of brain metabolic decline in preclinical Alzheimer’s disease

**DOI:** 10.1007/s00259-018-3933-3

**Published:** 2018-02-02

**Authors:** Tharick A. Pascoal, Sulantha Mathotaarachchi, Monica Shin, Ah Yeon Park, Sara Mohades, Andrea L. Benedet, Min Su Kang, Gassan Massarweh, Jean-Paul Soucy, Serge Gauthier, Pedro Rosa-Neto, for the Alzheimer’s Disease Neuroimaging Initiative

**Affiliations:** 1 Translational Neuroimaging Laboratory, The McGill University Research Centre for Studies in Aging, Douglas Hospital, McGill University, 6875 La Salle Blvd - FBC room 3149, Montreal, QC H4H 1R3 Canada; 2 Statistical Laboratory, University of Cambridge, Cambridge, UK; 3 Montreal Neurological Institute, Montreal, Canada; 4 PERFORM Centre, Concordia University, Montreal, Canada; 5 Alzheimer’s Disease Research Unit, The McGill University Research Centre for Studies in Aging, Douglas Hospital, McGill University, Montreal, Canada; 6 Department of Neurology and Neurosurgery, McGill University, Montreal, Canada

**Keywords:** Amyloid-PET, [^18^F]FDG PET, Preclinical Alzheimer’s disease, Phosphorylated tau

## Abstract

**Purpose:**

We aimed to determine the amyloid (Aβ) and tau biomarker levels associated with imminent Alzheimer’s disease (AD) - related metabolic decline in cognitively normal individuals.

**Methods:**

A threshold analysis was performed in 120 cognitively normal elderly individuals by modelling 2-year declines in brain glucose metabolism measured with [^18^F]fluorodeoxyglucose ([^18^F]FDG) as a function of [^18^F]florbetapir Aβ positron emission tomography (PET) and cerebrospinal fluid phosphorylated tau biomarker thresholds. Additionally, using a novel voxel-wise analytical framework, we determined the sample sizes needed to test an estimated 25% drugeffect with 80% of power on changes in FDG uptake over 2 years at every brain voxel.

**Results:**

The combination of [^18^F]florbetapir standardized uptake value ratios and phosphorylated-tau levels more than one standard deviation higher than their respective thresholds for biomarker abnormality was the best predictor of metabolic decline in individuals with preclinical AD. We also found that a clinical trial using these thresholds would require as few as 100 individuals to test a 25% drug effect on AD-related metabolic decline over 2 years.

**Conclusions:**

These results highlight the new concept that combined Aβ and tau thresholds can predict imminent neurodegeneration as an alternative framework with a high statistical power for testing the effect of disease-modifying therapies on [^18^F]FDG uptake decline over a typical 2-year clinical trial period in individuals with preclinical AD.

**Electronic supplementary material:**

The online version of this article (10.1007/s00259-018-3933-3) contains supplementary material, which is available to authorized users.

## Introduction

The preclinical stages of Alzheimer’s disease (AD) have become the main focus of therapeutic clinical trials given the assumption that better outcomes can be achieved with changes in the course of the disease before the appearance of cognitive symptoms [1, 2]. The International Working Group and the American Alzheimer’s Association have recently characterized preclinical AD as the presence of amyloid-β (Aβ) and tau abnormalities in cognitively normal persons [1]. Although these individuals show greater rates of disease progression than cognitively normal biomarker-negative individuals, most of them remain stable over typical clinical trial periods [1, 3]. Therefore, a critical next step proposed by the working groups was to identify, from among individuals with preclinical AD, those with the highest likelihood of disease progression within time frames acceptable for clinical trial designs taking into account financial, medical, and ethical considerations [1].

Its slow rate of change makes the use of cognition as the primary outcome of clinical trials in individuals with preclinical AD difficult because it leads to prohibitively high sample sizes and long follow-up times [4]. Hence, surrogate measurements of disease progression using established biomarkers of neurodegeneration might provide a useful alternative for such trials [5]. In fact, few studies have tested changes in structural magnetic resonance imaging (MRI) as a possible surrogate marker for preclinical AD clinical trials. For example, a recent study by the Alzheimer’s Disease Neuroimaging Initiative (ADNI) has suggested that to test for structural changes on MRI in a population of individuals with preclinical AD enriched on the basis of Aβ and tau biomarker positivity, sample size estimates are prohibitively large [3]. In this regard, changes in [^18^F]fluorodeoxyglucose ([^18^F]FDG) uptake have been suggested for following disease progression and monitoring therapeutic effects [6–11]. Indeed, [^18^F]FDG positron emission tomography (PET) is one of the most important biomarkers of AD with applications in the research and clinical settings. However, the characteristics of [^18^F]FDG uptake as a surrogate marker of preclinical AD in clinical trials are not fully known. Indeed, the inclusion of cognitively normal individuals harbouring Aβ and tau, and with abnormal [^18^F]FDG uptake could be argued as an interesting strategy for these trials, since such individuals have a higher probability of developing further neurodegeneration and cognitive symptoms [1]. However, it is reasonable to suggest that the absence of baseline neurodegeneration might offer a more favourable pathophysiological scenario for preventive therapies aiming to mitigate disease progression [2].

Recent literature indicates that Aβ and tau pathologies may arise independently and, at some pathophysiological point, synergistically potentiate imminent neurodegeneration in preclinical AD [2, 12–14]. Notably, this framework infers the existence of thresholds for Aβ and tau pathologies associated with the triggering of their deleterious synergy in potentiating neurodegeneration. However, the fact that the majority of Aβ-positive plus tau-positive individuals with preclinical AD remain pathophysiologically stable over long periods suggests that these thresholds are greater than their respective individual thresholds for abnormality [1]. Thus, determination of the Aβ and tau biomarker thresholds associated with imminent neurodegeneration might provide complementary information in individuals with preclinical AD destined to develop AD-related progression.

In this study, we tested the hypothesis that the combination of optimized Aβ and tau thresholds predictive of neurodegeneration might provide an alternative framework with a high statistical power for testing the efficacy of the emerging disease-modifying therapies. This framework has the potential to select preclinical individuals on the verge of AD-related progression, considering metabolic changes as indices of neurological decline.

## Materials and methods

### Participants

Data used in this study were obtained from the ADNI database (adni.loni.usc.edu). The ADNI was launched in 2003 as a public–private partnership led by the Principal Investigator Michael W. Weiner. The primary goal of ADNI has been to test whether serial MRI, PET, other biological markers, and clinical and neuropsychological assessment can be combined to measure the progression of mild cognitive impairment and early AD. For the present analysis, we selected 120 cognitively normal ADNI participants in whom cerebrospinal fluid (CSF) phosphorylated-tau (p-tau) was determined and [^18^F]florbetapir PET was performed at the same baseline visit, and [^18^F]FDG PET at the baseline visit and at the 2-year follow-up visit.

### CSF analysis

CSF p-tau and CSF Aβ were quantified with the INNO-BIA AlzBio3 immunoassay using the multiplex xMAP Luminex platform (Luminex Corp, Austin, TX). The CSF data used in the analysis were selected from the ADNI files “UPENNBIOMK5-8.csv”, and all the values for each subject were obtained from the same file. The standard published ADNI threshold for p-tau abnormality of >23 pg/ml was used in this analysis [15, 16]. Further details of methods for CSF acquisition and quantification can be found at www.adni-info.org.

### MRI/PET

MRI and PET acquisitions followed the ADNI protocols (http://adni.loni.usc.edu/methods). The MRI T1-weighted images underwent nonuniformity correction, brain masking and segmentation using the Brain Extraction based on nonlocal Segmentation Technique [17]. The T1-weighted images were then processed using the CIVET image-processing pipeline and registered using a nine-parameter affine transformation and nonlinearly spatially normalized to the MNI 152 template [18]. PET images were smoothed using a volumetric gaussian kernel with a full-width at half-maximum of 8 mm. Subsequently, linear registration and nonlinear normalization to the MNI 152 template were performed with the linear and nonlinear transformation derived from the automatic PET to MRI transformation and the individual’s anatomical MRI coregistration. [^18^F]Florbetapir and [^18^F]FDG standardized uptake value ratio (SUVR) maps were generated using the cerebellum grey matter and the pons as reference regions, respectively [13]. Global PET SUVR values for each subject were estimated from the precuneus, prefrontal, orbitofrontal, parietal, temporal, and cingulate cortices. In our pipeline, 30% of controls were Aβ-positive using a standard [^18^F]florbetapir SUVR threshold of 1.15, which is consistent with ADNI publications [19, 20]. Further details regarding our image-processing pipeline can be found elsewhere [13, 14].

### Statistical methods

#### Threshold analysis

We determined the thresholds predictive of metabolic decline by modelling Δ[^18^F]FDG ($\left(\frac{\mathrm{SUVR}\ \mathrm{Follow}\ \mathrm{up}-\mathrm{SUVR}\ \mathrm{Baseline}}{\mathrm{SUVR}\ \mathrm{Baseline}}\right)\ast \frac{100}{\Delta  \mathrm{Time}}$) as a function of baseline [^18^F]florbetapir and p-tau thresholds in regions of interest. The regions were segmented using the coordinates in the MNI ICBM atlas [21], and the threshold analysis was performed using GraphPad Prism 6.0 software. *R*-squared and the *F*-test were used to evaluate the goodness-of-fit, while the Akaike information criterion (AIC) was used to compare linear and nonlinear functions. Nonlinear associations were assumed to be sigmoidal, as discussed previously [22], and were formulated as follows:

$$ Y=a+\left(\frac{b}{1+{\mathrm{e}}^{\frac{-\left(X-c\right)}{d}}}\right) $$

In this equation, *Y* is the Δ[^18^F]FDG, *X* is the biomarker threshold, and *a*, *b*, *c* and *d* are the parameters of the fitting, where *a* corresponds to the lower asymptote of the Δ[^18^F]FDG at the unit of *Y*, *b* corresponds to the total change in Δ[^18^F]FDG as a function of biomarkers (value between the lower and the upper asymptote) at the unit of *Y*, *c* is the Δ[^18^F]FDG at the inflection point of the curve at the unit of *X*, and *d* represents the curve steepness. The biomarker threshold for imminent metabolic decline was defined as the value where the curvatures changed sign (*X*(*c*)). This point was assumed as the magnitude that needs to be reached by the biomarker for the determination of a significant 2-year metabolic decline.

#### Group comparison analysis

Groups were compared using analysis of covariance (ANCOVA) using the R statistical software package version 3.1 to test for significant differences in metabolic decline between biomarker groups and also in demographic differences between biomarker groups for continuous variables, whereas the chi-squared test was used for the categorical ones. The *P* values are presented after correction for multiple comparisons testing using Bonferroni correction at a significance level of 0.05, and differences in metabolic decline among the biomarker groups were further adjusted for age, gender, education, and *APOE-ε4* carrier status.

#### Voxel-wise sample size calculation

A voxel-wise power analysis was performed using MATLAB 15a software with a novel analytical tool adapted to assess the sample size necessary for a clinical trial testing for a drug effect on Δ[^18^F]FDG uptake at every brain voxel [23]. To the best of our knowledge, this is the first PET study in which voxel-wise power calculations have been performed. The sample calculations estimated the number of subjects required to detect 25% slowing in Δ[^18^F]FDG uptake for a hypothetical disease-modifying therapy versus placebo with 80% of power at a 5% level [24, 25]. The analysis was performed with a well-described formula across the biomarker groups [24–27]:

$$ \mathrm{sample}\ \mathrm{size}\ \left(\mathrm{voxel}\ \left(\mathrm{x},\mathrm{y},\mathrm{z}\right)\right)={\left(\partial +\delta \right)}^2\ast \frac{2{\upsigma}^2}{{\left(\Delta  \mu \ast \beta \right)}^2} $$

We used *∂* = 0.842 to power at 80% and *δ* = 1.96 to test a significance of 5%; ∆*μ* is the average percentage of change in the signal intensity of [^18^F]FDG; *β* is the drug effect of 0.25 to reflect the difference in [^18^F]FDG uptake between drug and placebo groups; *σ* is the standard deviation (SD) of [^18^F]FDG uptake changes, and (*x*,*y*,*z*) is the coordinate of each voxel. Importantly, the parametric images showed the regions with a significant metabolic decline over 2 years after correction for multiple comparisons testing using a false discovery rate at *P* < 0.001.

## Results

### Threshold analysis

**Fig. 1:**
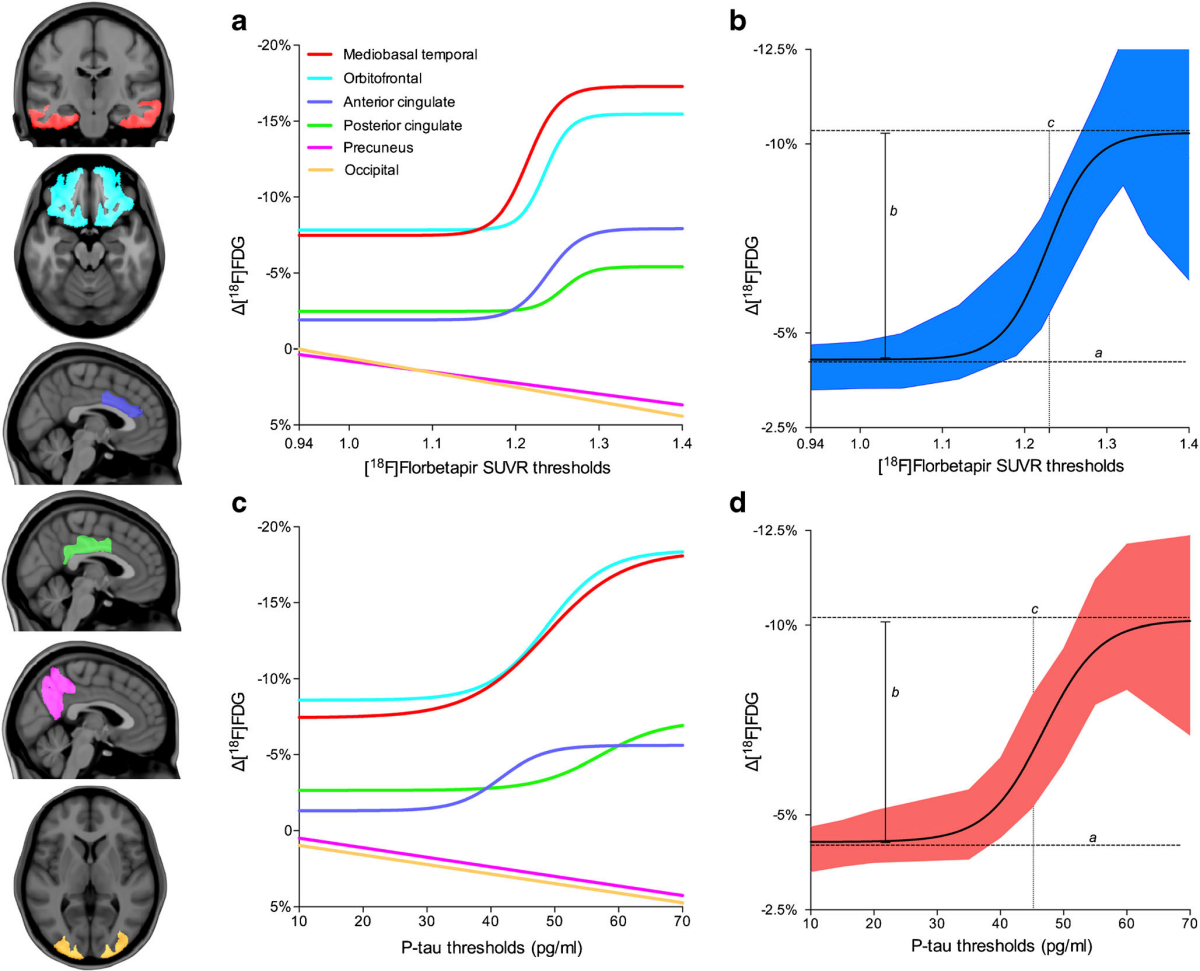
Aβ and tau thresholds associated with imminent metabolic decline are higher than their standard thresholds for biomarker abnormality. Curves represent changes in [^18^F]FDG uptake over 2 years within anatomically segregated clusters as a function of baseline [^18^F]florbetapir SUVR and CSF p-tau thresholds. **a** As a function of [^18^F]florbetapir thresholds, [^18^F]FDG uptake decline fits a sigmoidal curve in the mediobasal temporal cortex (*R*^2^ = 0.98), the orbitofrontal cortex (*R*^2^ = 0.97) the anterior cingulate cortex (*R*^2^ = 0.97) and the posterior cingulate cortex (*R*^2^ = 0.97). **b** In the average clusters, the inflection point of [^18^F]FDG uptake decline was at [^18^F]florbetapir SUVR 1.228 (*R*^2^ = 0.98, 95% CI 1.205–1.253). **c** As a function of CSF p-tau thresholds, [^18^F]FDG uptake decline fitted a sigmoidal curve in the mesiobasal temporal cortex (*R*^2^ = 0.97), the orbitofrontal cortex (*R*^2^ = 0.96), the anterior cingulate cortex (*R*^2^ = 0.98) and the posterior cingulate cortex (*R*^2^ = 0.94). **d** The inflection point of the sigmoidal curve in the averaged clusters was at a CSF p-tau of 45 pg/ml (95% CI 43.72–47.9). Notably, clusters in the precuneus and occipital lobe did not have any linear or sigmoidal association with biomarker thresholds. In the curves, *a* corresponds to the lower asymptote, *b* corresponds the total change in Δ[^18^F]FDG uptake, and *c* is the Δ[^18^F]FDG uptake at the inflection point of the curve

A sigmoidal curve was the best fit to represent the decline in [^18^F]FDG uptake over 2 years as a function of [^18^F]florbetapir thresholds in the mediobasal temporal cortex (*R*^2^ = 0.98, AIC 11.83 with probability of correctness, PC, 96.57% versus 3.43% for the linear model), orbitofrontal cortex (*R*^2^ = 0.97, AIC 17.11, PC 99.98%), anterior cingulate cortex (*R*^2^ = 0.97, AIC 1.86, PC cortex 71.68%), and posterior cingulate cortex (*R*^2^ = 0.97, AIC 6.43, PC 96.13%). Based on the inflection point of the preferred sigmoidal model, a curve-fitting threshold analysis on averaged clusters (*R*^2^ = 0.98, AIC 5.26, PC 93.28%) revealed that the optimal [^18^F]florbetapir threshold predictive of imminent metabolic decline was 1.228 (95% CI 1.205–1.253, 1 SD higher than the standard threshold; Fig. 1a, b; Supplementary Table 1). Interestingly, although [^18^F]florbetapir SUVR was highly correlated with CSF Aβ (Spearman’s rho = 0.68, *P* < 0.0001), CSF Aβ thresholds did not significantly model the declines in [^18^F]FDG uptake in the above regions in our population.

A sigmoidal curve was the best fit to represent declines in [^18^F]FDG uptake as a function of p-tau thresholds in the mesiobasal temporal cortex (*R*^2^ = 0.97, AIC 7.53, PC 97.74%), the orbitofrontal cortex (*R*^2^ = 0.96, AIC 7.88, PC 98%), the anterior cingulate cortex (*R*^2^ = 0.98, AIC 23, PC >99%), the posterior cingulate cortex (*R*^2^ = 0.94, AIC 5.15, PC >96%), and averaged clusters (*R*^2^ = 0.94, AIC 8, PC 98.23%). Curve-fitting threshold analysis on averaged clusters revealed an inflection point of 45 pg/ml (95% CI 43.72–47.9 pg/ml, 1.3 SD higher than the standard threshold; Fig. 1c, d; Supplementary Table 1).

**Fig. 2:**
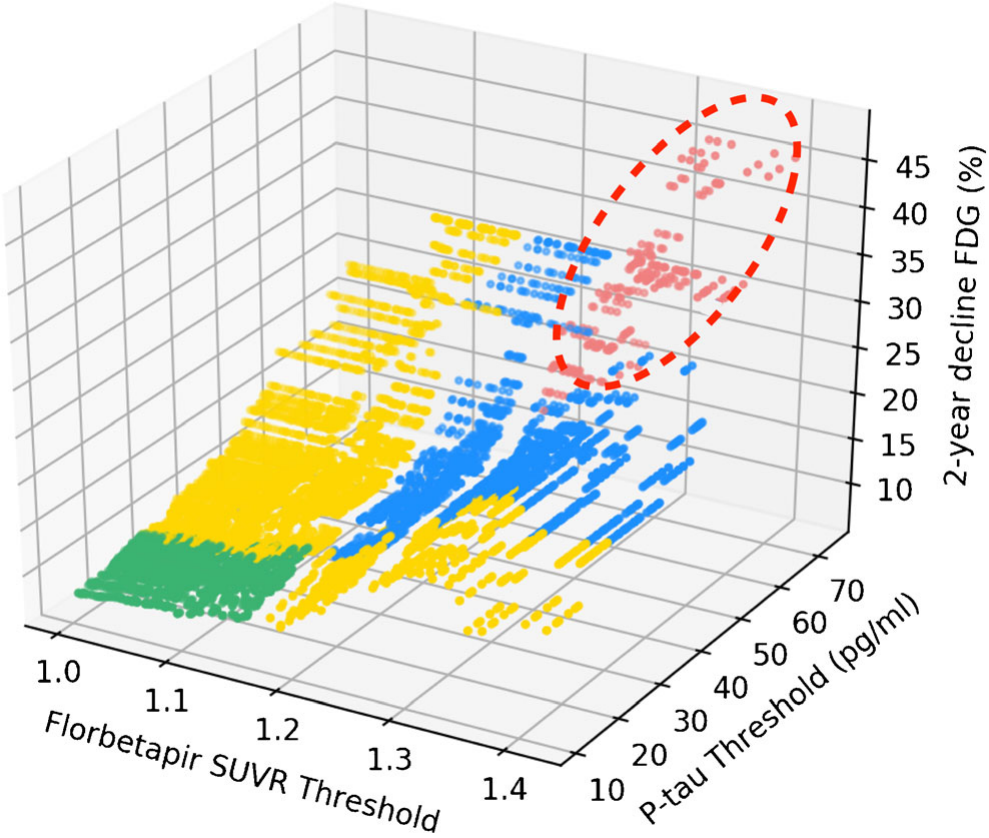
Preclinical AD groups with progressively higher Aβ and tau thresholds, with both biomarker levels greater than those for imminent metabolic decline, had progressively higher rates of [^18^F]FDG hypometabolism. The *dots* in the 3D plot represent the mean metabolic decline in clusters over 2 years in Aβ-positive plus p-tau-positive groups segregated using all possible combinations (*n* = 14,400) of threshold values for [^18^F]florbetapir SUVR and CSF p-tau. The groups with both biomarker thresholds lower than the standard values (*green*), with only one biomarker threshold higher than the standard thresholds (*yellow*) and with both biomarker thresholds higher than the standard values with at least one lower than the threshold (*blue*) for imminent metabolic decline did not show a significant 2-year decline in [^18^F]FDG uptake. On the other hand, Aβ-positive plus p-tau-positive groups segregated using both thresholds equal to or higher than the proposed thresholds showed progressively higher rates of metabolic decline with the progressive increase in the biomarker threshold values (*red*)

In addition, we plotted Δ[^18^F]FDG uptake inside clusters in [^18^F]florbetapir-positive plus p-tau-positive groups segregated using all possible combinations of thresholds for both biomarkers (*n* = 14,400). The 3D plot confirms that individuals with preclinical AD defined using progressively higher biomarker thresholds, with both biomarker levels greater than the thresholds for imminent metabolic decline, had progressively higher rates of Δ[^18^F]FDG hypometabolism (Fig. 2).

### Group comparison analysis

Applying the new criteria for preclinical AD with standard thresholds [1], out of 120 individuals, 24 (20%) were biomarker-negative, 63 (53%) were at risk of AD (only one biomarker abnormality), while 33 (27%) showed preclinical AD with both Aβ and p-tau abnormalities. Using the proposed combination of [^18^F]florbetapir (SUVR >1.228) and CSF p-tau (>45 pg/ml) thresholds predictive of imminent metabolic decline, out of 33 individuals with preclinical AD, 17 (14%) had both biomarkers above the thresholds. Importantly, voxel-wise comparison revealed that all the above biomarker groups did not show any significant differences in [^18^F]FDG uptake at the baseline visit. Demographics and key sample characteristics of the individuals of the population across biomarker groups are summarized in Table 1.

**Table 1:** Demographics and key characteristics of the population in each biomarker group

Characteristic	Biomarker-negative	At risk of AD	Preclinical AD below thresholds	Preclinical AD above thresholds	*P* value
No. of subjects	24	63	16	17	
Age (years), mean (SD)	75.1 (7.4)	73.9 (6.1)	74.9 (6.2)	78.6 (5.1)	0.37
Male, *n* (%)	14 (58)	35 (55)	7 (44)	7 (42)	0.3
Education (years), mean (SD)	17.7 (2.5)	16.3 (2.9)	15.6 (2.4)	16.7 (2.5)	0.6
MMSE score, mean (SD)					
Baseline	29.1 (1.4)	29.2 (0.96)	28.9 (0.97)	29 (0.92)	0.81
Follow-up	29 (1.6)	29 (1.1)	28.4 (1.9)	28.6 (1.3)	0.27
*APOE-ε4* carrier, *n* (%)	1 (4)	17 (27)	6 (38)	6 (35)	0.06
P-tau (pg/ml), mean (SD)	19.3 (2.9)	35.8 (14.8)*	36.5 (8.9)*	56.4 (15.8)*	<0.001
[^18^F]Florbetapir (SUVR), mean (SD)	1 (0.03)	1.12 (1)*	1.25 (0.08)*	1.35 (0.07)*	<0.001
Follow-up (months), mean (SD)	24.3 (0.98)	23.8 (0.9)	24.1 (1.8)	24 (0.7)	0.53
Diagnostic at follow-up visit, *n* (%)					
Cognitively normal	22 (91)	55 (87.5)	14 (88)	12 (71)	**–**
Mild cognitive impairment	2 (9)	7 (11)	2 (12)	5 (29)	0.2
Dementia	0	1 (1.5)	0	0	**–**

The values in each group were compared using analysis of covariance for each variable except gender, *APOE-ε4* carrier status, and diagnostic at follow-up, for which the chi-squared test was used.

*MMSE* Mini-Mental State Examination

*P < 0.05, vs. biomarkers-negative group, post-hoc analysis

**Fig. 3:**
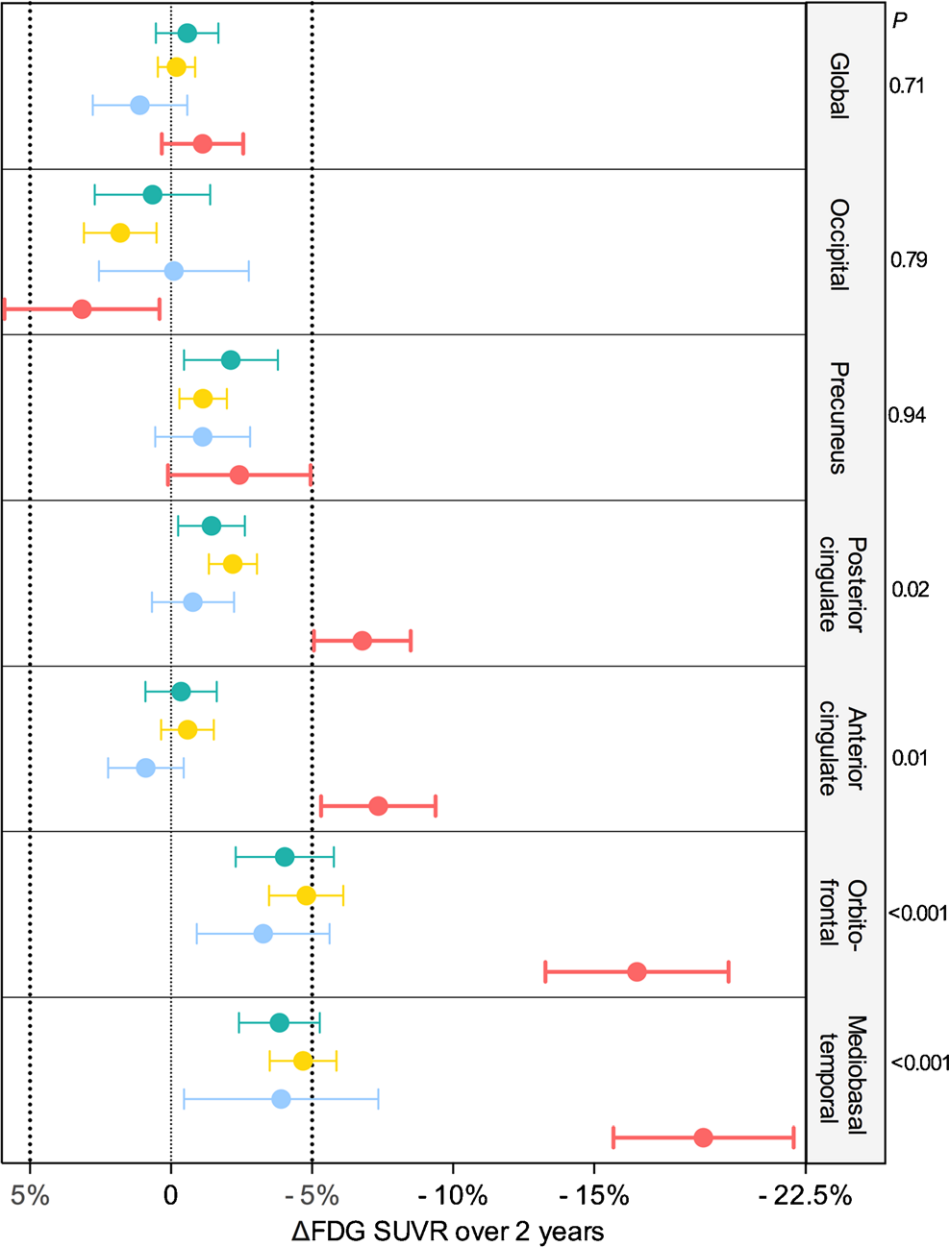
In our cognitively normal population of individuals with preclinical AD, those with Aβ and tau above the optimized thresholds showed the highest rates of 2-year AD-related metabolic decline. ANCOVA confirmed that these individuals (with [^18^F]florbetapir SUVR >1.23 and p-tau >45 pg/ml; *n* = 17, *red*) showed the highest 2-year rates of [^18^F]FDG uptake decline in the mediobasal temporal, orbitofrontal, and cingulate cortices. It is important to emphasize that individuals with preclinical AD and at least one of the biomarkers below the proposed threshold (*n* = 16, *blue*) showed declines in [^18^F]FDG uptake not different from biomarker-negative individuals (*n* = 24, *green*) and those at risk of AD (*n* = 63, *yellow*). The analysis was adjusted for age, gender and *APOE-ε4* carrier status, with Bonferroni correction at a significance level of 0.05

ANCOVA of the average decline in [^18^F]FDG uptake in each region confirmed that individuals with [^18^F]florbetapir plus p-tau levels above the proposed thresholds showed the highest rates of [^18^F]FDG decline in the mediobasal temporal, orbitofrontal, anterior cingulate and posterior cingulate cortices in our population (Fig. 3). The biomarker groups did not show any differences in average [^18^F]FDG uptake at baseline in any region of interest. Interestingly, in the precuneus and the occipital lobe, there were no significant differences in the decline in [^18^F]FDG uptake across the biomarker groups.

### Voxel-wise sample size calculation

**Fig. 4:**
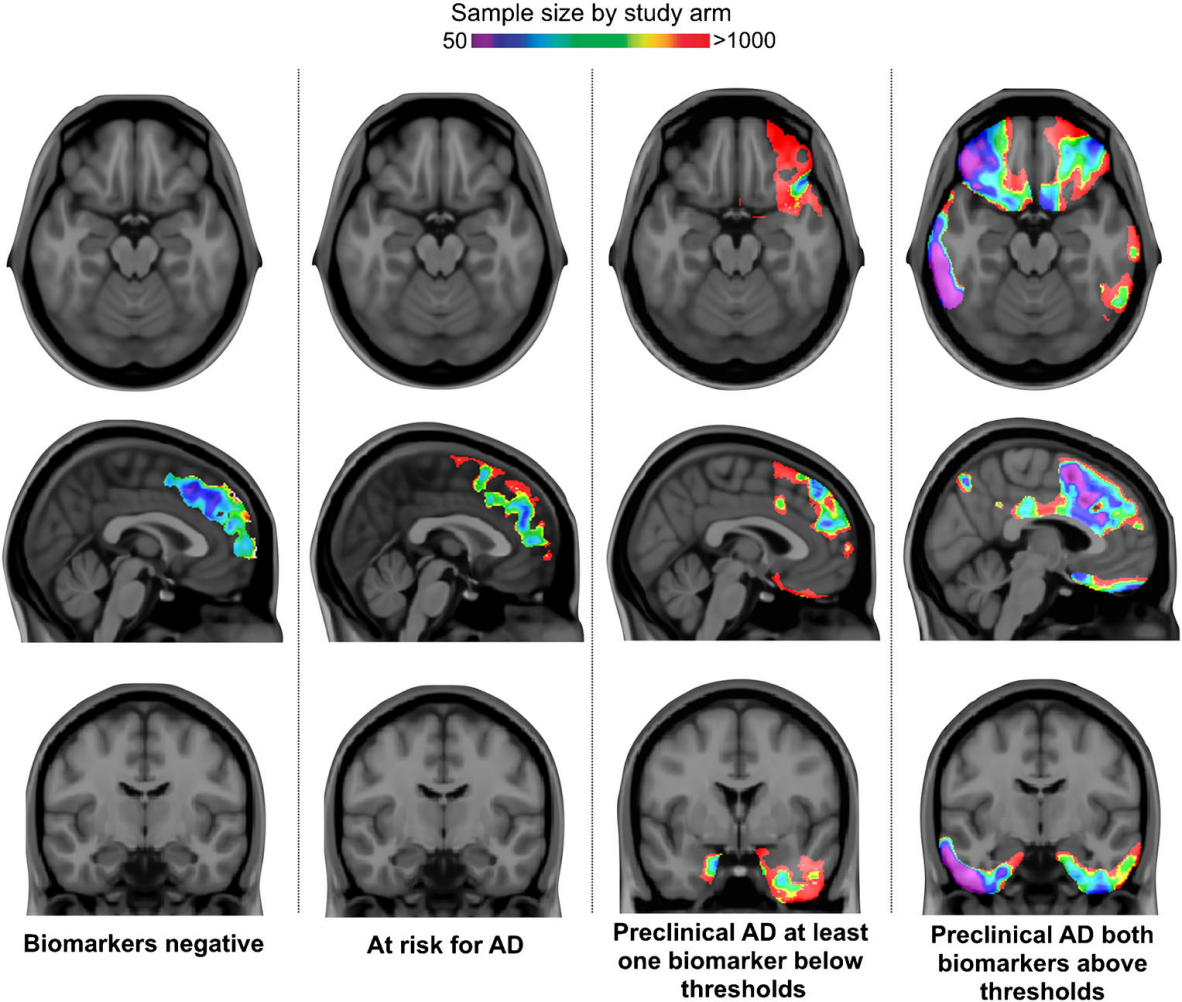
Voxel-wise power analysis confirmed that defining preclinical AD according to the thresholds predictive of imminent metabolic decline offers a robust framework with a high statistical power for population enrichment of clinical trials using regional Δ[^18^F]FDG uptake as a surrogate marker. The parametric maps represent the voxel-wise sample size calculations overlain on structural MRI scans showing the regions with a significant decline in [^18^F]FDG uptake in each biomarker group after multiple comparison correction (false discovery rate at *P* < 0.001). The maps confirm that individuals with both biomarkers above the threshold of imminent metabolic decline provide the best framework to test a 25% drug effect on changes in [^18^F]FDG uptake in large clusters in the mediobasal temporal, cingulate, and orbitofrontal cortices, requiring a sample as small as 50 individuals per trial arm

A voxel-wise power analysis – free of anatomical assumptions - further confirmed that identifying individuals with both biomarkers above the proposed thresholds is the best approach to population enrichment in therapeutic trials using changes in voxel-wise [^18^F]FDG uptake over 2 years in large clusters in the orbitofrontal, anterior and posterior cingulate, and mediobasal and lateral temporal cortices as a surrogate marker of preclinical AD requiring as few as 50 individuals per trial arm (Fig. 4).

Additionally, to ensure that the results of our voxel-wise power calculation were not affected by any issues related to the voxel-wise approach, we averaged the SUVR values inside segregated regions and performed the same analyses for each region using these average SUVRs as the outcome (Supplementary Table 2). Using the average Δ[^18^F]FDG uptake in the mediobasal temporal cortex as a surrogate marker, a study population enriched by including those with both biomarkers above the proposed thresholds ([^18^F]florbetapir SUVR >1.228 and p-tau >45 pg/ml) would require 87 individuals with preclinical AD per trial arm to test a 25% drug effect (80% of power at a 5% level) over 2 years. On the other hand, a clinical trial using the concept of preclinical AD with standard thresholds ([^18^F]florbetapir SUVR >1.15 and p-tau >23 pg/ml) would require 416 individuals per arm, whereas a trial without a population enrichment strategy would require 738 individuals per arm.

## Discussion

In summary, our results suggest that the Aβ and tau thresholds associated with imminent AD-related metabolic decline in individuals with preclinical AD are higher than their respective thresholds for abnormality. In addition, we showed that the use of these thresholds and the use of voxel-wise changes in [^18^F]FDG uptake as a surrogate marker as inclusion criteria provide an alternative framework with a high statistical power for 2-year clinical trials in preclinical AD. Overall, we propose the new concept of Aβ and tau thresholds for imminent neurodegeneration as a valuable approach to population enrichment in clinical trials in individuals with preclinical AD and a high probability of developing AD-related neurodegeneration within a short time. Specifically, we demonstrated that a 2-year clinical trial in cognitively normal individuals using brain Aβ plus CSF p-tau thresholds more than one SD higher than their standard values together with regional [^18^F]FDG uptake as a surrogate marker would require as few as 100 individuals to test a hypothetical 25% drug effect. These results contrast with a recent study performed by ADNI in individuals with preclinical AD defined as those with Aβ plus tau positivity which showed that a 2-year clinical trial measuring changes in cognition or structural MRI would require more than 2,000 individuals to test a hypothetical 25% drug effect with 80% power [3].

It is important to emphasize that the thresholds proposed here are based on dynamic biomarker changes and indicate metabolic decline over short time frames, rather than the presence or absence of pathological proteinopathies, which are best assessed with post-mortem correlations [28]. It is also important to mention that in our understanding the term “biomarker abnormality” refers to the presence of brain pathology and should be defined by thresholds that are better associated with post-mortem studies. Within the biomarker abnormality spectrum, we suggest that the existence of thresholds for imminent disease progression will identify from among individuals with biomarker abnormalities those with the highest probability of progression to a given outcome.

Since the proposed model optimizes neurodegeneration as a function of Aβ and tau levels, we may argue that this model has immediate applicability to provide a framework with a high statistical power for use with the emerging anti-tau and anti-amyloid therapies. Interestingly, the highest rates of metabolic decline shown by individuals with preclinical AD were found in the limbic structures within the cingulate, orbitofrontal, and medial and basal temporal cortices. Importantly, this pattern of metabolic dysfunction is not considered part of normal ageing [29, 30]. In fact, metabolic decline in the limbic structures has been proposed as an early abnormality associated with AD [31, 32]. In addition, the fact that this brain circuit is affected by tangles or plaques [29, 30], rather than by the normal ageing process [33–35], further supports the notion that cognitively normal subjects with these baseline biomarker signatures are on the AD pathway. Interestingly, in contrast with [^18^F]florbetapir, CSF Aβ levels failed to model imminent metabolic decline in our population, which is in line with previous observations showing that Aβ PET depicts metabolic decline better than CSF Aβ in this preclinical AD population [13]. Although CSF Aβ well-represents the disease status, brain fibrillar Aβ deposition seems to be better associated with imminent disease progression [13, 36].

Declines in [^18^F]FDG uptake as a function of the hallmark AD proteins were best described by a sigmoidal rather than a linear association. This pattern of relationship is consistent with the most accepted models of AD progression [22], which assume “ceiling effects” for Aβ and tau. Therefore, our results further emphasize that this ceiling effect should be considered in studies testing the relationship between these proteinopathies and downstream neurodegeneration. For example, studies using linear functions for testing the association between proteins and brain hypometabolism in AD patients might not show any correlation [13], since it is likely that most demented patients have already reached the plateau of the relationship.

Methodological aspects limit the external validity of the present results. Our population was composed of self-selected individuals and might not represent the general elderly cognitively normal population. Therefore, it would be highly desirable to replicate our results in a larger population-based study. However, it is important to mention that the use of a conservative multiple comparison correction threshold of 0.001 helped to avoid false-positives results in our analysis. It is also important to emphasize that change in cognition is always the most desirable outcome for a therapeutic clinical trial aiming to mitigate AD progression. However, due to the methodological limitations in the use of cognition in individuals with preclinical AD and the advances in in vivo biomarkers, increasing our understanding of the use of brain imaging in the assessment of AD progression is of paramount importance. Importantly, individuals with preclinical AD in this study were considered those with normal cognition but Aβ and tau abnormalities rather than those who would certainly have developed dementia over time. The 2-year follow-up was not sufficient to draw definite conclusions regarding clinical progression from cognitively normal to dementia.

Partial volume correction did not translate into differences in our final results. Therefore, we present the PET data without corrections for volume effects. Biomarker thresholds are invariably subject to idiosyncrasies and as such might vary slightly depending on the analytical method used. However, in this study, biomarker levels predictive of rapid metabolic decline were at least one SD higher than the threshold for the presence of brain pathology in an elderly cognitively normal population. Notably, the thresholds associated with imminent disease progression would invariably be expected to be higher than those used to determine an abnormal biomarker status. It is important to mention that the use of a hypothetical drug effect of 25% used in our analysis was the same as that used in previous studies in AD [3, 24, 25, 27]. Although study population enrichment with two biomarker modalities might potentially have a high economic cost, it is important to emphasize that an increasingly large number of observational studies using multiple biomarkers for Aβ and tau have indicated that this approach could serve as a screening tool to select individuals for therapeutic trials. Despite the current lack of an effective disease-modifying therapy for AD, the promising recent results with anti-Aβ drugs such as aducanumab suggest the need for sensitive methods to detect disease progression.

To conclude, our results show that Aβ and tau biomarker thresholds associated with imminent neurodegeneration are higher than their respective thresholds for abnormality. In addition, the determination of these thresholds may provide complementary information for selecting individuals with preclinical AD who will most likely show pathophysiological progression over time frames compatible with clinical trials.

## Electronic supplementary material

Supplementary Table 1 (DOCX 97 kb)

Supplementary Table 2 (DOCX 60 kb)
