# Supplementary material for: Amyloid and tau signatures of brain metabolic decline in preclinical Alzheimer’s disease
Source: Eur J Nucl Med Mol Imaging. 2018 Feb 2;45(6):1021–30. doi: 10.1007/s00259-018-3933-3 (PMC5915512; doi:10.1007/s00259-018-3933-3)
Supplement: Supplementary file 1 — (DOCX 97 kb) [file 259_2018_3933_MOESM1_ESM.docx]

**Supplementary Table 1. [^18^F]FDG uptake decline as a function of baseline [^18^F]florbetapir SUVR and CSF p-tau thresholds best fitted a sigmoidal function in AD-related regions.**

| [^18^F]Florbetapir SUVR | | | | | | | |
| --- | --- | --- | --- | --- | --- | --- | --- |
| Δ[^18^F]FDG | **Probability of correctness (%)** | | | **R^2^** | | **AIC** | ***P* value** |
|  | **Linear** | | **Sigmoid** | **Linear** | **Sigmoid** |  |  |
| Mediobasal temporal | | 3.43 | 96.57 | 0.85 | 0.98 | 11.83 | <0.001 |
| Orbitofrontal | | 0.02 | 99.98 | 0.77 | 0.97 | 17.11 | <0.001 |
| Anterior Cingulate | | 28.32 | 71.68 | 0.62 | 0.97 | 1.857 | <0.001 |
| Posterior Cingulate | | 3.87 | 96.13 | 0.83 | 0.94 | 6.432 | <0.001 |
|  | | | | | | | |
| CSF p-tau | | | | | | | |
| Δ[^18^F]FDG | **Probability of correctness (%)** | | | **R^2^** | | **AIC** | ***P* value** |
|  | **Linear** | | **Sigmoid** | **Linear** | **Sigmoid** |  |  |
| Mediobasal temporal | | 2.26 | 97.74 | 0.89 | 0.97 | 7.531 | <0.001 |
| Orbitofrontal | | 2 | 98 | 0.86 | 0.96 | 7.885 | <0.001 |
| Anterior Cingulate | | 0.6 | 99.4 | 0.87 | 0.98 | 23 | <0.001 |
| Posterior Cingulate | | 3.8 | 96.2 | 0.77 | 0.94 | 5.15 | <0.001 |

Sigmoidal model was the better fit for mediobasal temporal, orbitofrontal, anterior and posterior cingulate cortices when compared with the linear analyses. No linear or sigmoidal functions significantly fitted in the precuneus and occipital cortices. AIC=Akaike information criterion; CSF=cerebrospinal fluid, FDG=fluorodeoxyglucose, p-tau=phosphorylated tau, SUVR=standardized uptake value ratio.
