# Supplementary material for: Amyloid and tau signatures of brain metabolic decline in preclinical Alzheimer’s disease
Source: Eur J Nucl Med Mol Imaging. 2018 Feb 2;45(6):1021–30. doi: 10.1007/s00259-018-3933-3 (PMC5915512; doi:10.1007/s00259-018-3933-3)
Supplement: Supplementary file 2 — (DOCX 60 kb) [file 259_2018_3933_MOESM2_ESM.docx]

**Supplementary table 2. Required sample sizes per study arm for a 2-year clinical trial using changes in [^18^F]FDG SUVR uptake in averaged regions-of-interest as a surrogate variable.**

| Enrichment strategy | Mediobasal temporal | Orbito-frontal | Posterior cingulate | Anterior cingulate | Global |
| --- | --- | --- | --- | --- | --- |
| None | 738 | 987 | 2300 | 7330 | ^*^No decline |
| Aβ+ | 567 | 1162 | 1739 | 4150 | ^*^No decline |
| P-tau+ | 605 | 761 | 1728 | 5965 | ^*^No decline |
| Aβ+/ p-tau+ | 416 | 724 | 1356 | 2232 | ^*^No decline |
| Aβ+/ p-tau+ (above thresholds) | 87 | 369 | 581 | 542 | 4520 |

Aβ=amyloid-β, FDG=fluorodeoxyglucose, p-tau=phosphorylated tau, SUVR=standardized uptake value ratio. * No decline in mean [^18^F]FDG uptake over 2 years follow-up.
